# Quo Vadis, Cochrane Collaboration?

**DOI:** 10.1002/cre2.176

**Published:** 2019-02-27

**Authors:** Asbjorn Jokstad

**Affiliations:** ^1^ Department of Clinical Dentistry, Faculty of Health of Health Sciences UiT The Arctic University of Norway

The Cochrane Collaboration became a registered British charity in 1995; hence a few years after the first Cochrane centre opened in November 1992 in U.K. (U.K. Charity Commission, 2019a). Public information reveals that the charity maintains a staff of 64 employees and that the annual income and endowments to the central organization in 2017 total 8.7 million GBP and it retains assets over 7 million GBP (U.K. Charity Commission, 2019b). The significant bulk of the income is publication royalties from John Wiley and sons. A further 15.6 million GBP funded a global network of more than 38 thousand collaborators in 120 countries received from national governments, international governmental and non‐governmental organizations, universities, hospitals, private foundations, and personal donations (https://www.cochrane.org/about‐us/our‐funders‐and‐partners). It is perplexing and sad to read that this once idealistic organization is currently undergoing a crisis and tormented by multiple escalating controversies (Newman, 2019).

Upon scrutiny of recent articles, letters and multiple blogs, the issues that are being raised are about centralization, corporatization, governance and perceived conflicts of interest. Several allegations have been claimed of a near‐Orwellian‐like organizational culture introduced in 2015 to bolster the new brand “Cochrane” (http://fabrikbrands.com/portfolio/cochrane‐branding). The central office of the charity mandated all to stop referring to the full name of the charity, i.e., Cochrane Collaboration, with the notion that by only referring to “Cochrane” “..make things clear and consistent and maximize impact” (Cochrane brand, 2019a). The latest brand guidelines is a 144‐page document that details what to do and say and what not, and with suggestions on how to describe the charity, its history and its current efforts and visions with many beautiful words (Cochrane brand, 2019b).

It seems to undersigned that this initially idealistic charity has contracted some form of Icarus syndrome prompted by seduction to generate substantial revenues rather than strengthening the actual value of the offered products, i.e., the access to the databases of RCTs and systematic reviews (SR) and quality assurance of the latter category. Moreover, from a research ethics perspective, one may question why the Cochrane Collaboration has still not established a policy not to include in SRs primary studies that fail to report an approval by an ethics committee or institutional review board (Jokstad, 2017).

The charity has repeatedly stated that one of the main goals is to make evidence accessible and useful to everybody, everywhere in the world. However, this is not compatible with the current position on open access (OA) which is: “ … maintaining and expanding Cochrane Library revenues” (Cochrane, 2019). The initiative for OA launched by Science Europe in September 2018 seems not to be on the agenda within the Cochrane Collaboration, as judged by a search on their website. (Alternatively, the website search index has not been re‐indexed lately). True, since 2013 a hybrid (“green”) access policy has been in place, i.e., a 12 months embargo followed by open access, alternatively an option for the authors of SRs to pay an article‐processing charge (APC) of $5000 for full (“gold”) access (Cochrane Open Access, 2019). This arrangement does not set the charity apart and is analogue to the practices of most commercial publishers. Moreover, the APC is higher than the APC of most commercial publishers. The multiple “news” infrequently on Cochrane.org/news about this and that country now having free access to Cochrane SRs, usually complemented by the number of millions inhabiting the specified country, is not because of the charity, but rather because the contracted publisher of the Cochrane SRs, John Wiley & Sons, endorse the Hinari initiative established by WHO in 2001 (WHO, 2019).

**Figure 1:**
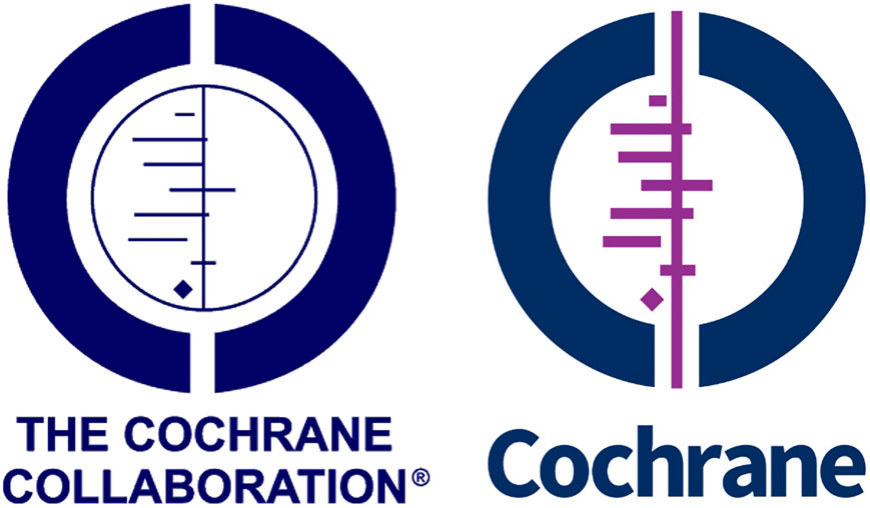
The old and the rebranded logo of the Cochrane Collaboration (since Jan 30, 2015). The graph is designed to illustrate the human costs that can result from failure to prepare systematic, up‐to‐date reviews of controlled trials of health care, such as the effects of prenatal corticosteroids on the likelihood of early neonatal mortality. Recent research may demand a bottom text: "Disclaimer: the findings shown in this logo pertain only to certain regions and patient populations."

The “value” of the Cochrane SRs is a reputation of comprehensiveness and objectivity, which encompasses a thorough search for trials and an impartial assessment of the identified evidence that include estimations of the likelihood of bias. They may perhaps be trustworthy, but they are not truths. Rather, they are best guesses, sometimes including a range of uncertainty. When it comes to the likelihood of being false, a meta‐analysis of small, inconclusive studies is statistically likely to have a positive predictive value (PPV, or false positive finding) that is below 50% under most premises (Ioannidis, 2005). Stated another way, the great majority of Cochrane SRs currently fall within this category, and the findings are therefore more likely to be false than not false. The forest plot logo of the charity itself is a good example of how one may become lured. The logo reflects a touching narrative about baby lives that could have been saved if gynecologists in the eighties had been aware of a handful of RCTs published starting from the early seventies (Figure 1). Since many have accepted this “truth”, WHO and NGOs committed to improving international social inequalities made several initiatives to encourage the use of prenatal corticosteroids for reducing morbidity and mortality after preterm birth. However, a paper in Lancet appeared in 2014 titled “Extreme caution is needed before scale‐up of antenatal corticosteroids to reduce preterm deaths in low‐income settings” (Azad & Costello, 2014), which seriously jolted the confidence of doing more good than harm. WHO convened very rapidly an expert group, which concluded that the generalisability of the available evidence demonstrate that a true state of clinical equipoise exists for this treatment option in low‐resource settings and that there was a clear need for more efficacy trials of ACS in these settings (Vogel et al., 2017). Also, the latest Cochrane SR on this topic added in the conclusions that “...the results may not be applicable to low‐resource settings with high rates of infections” (Roberts, Brown, Medley, & Dalziel, 2017). This message is quite different from the earlier conclusions like “No adverse consequences of prophylactic corticosteroids for preterm birth have been identified” in the 2000 update, and “A single course of antenatal corticosteroids should be considered routine for preterm delivery with few exceptions” concluded in the 2006 update. One pertinent remark is from the recognized founder of the charity, i.e., Iain Chalmers, who blogged: Should the Cochrane logo be accompanied by a health warning? (Chalmers, 2016). An alternative way of reorienting or educating readers would be to add the text below the logo stating: “Disclaimer: the findings shown in this logo pertain only to certain regions and patient populations”.

Upon reading the background history about the first SR on use of prenatal corticosteroids (www.histmodbiomed.org/sites/default/files/44848.pdf), one cannot help speculating whether the antagonists on the safety of corticosteroids at the time were “biased” because they had worked mostly within socially deprived communities or only with healthier patients within more robust health care systems. Regardless, between the two opposite beliefs of what was “trustworthy” at the time, one continued to prevail (Crowley, 1981), forming also the basis of the Cochrane Collaboration logo, while the other data and their interpretations were relegated to obscurity (Sachs, 1981) – in retrospect perhaps unjustly based on the recent clinical findings. In sum, it is not possible to generate numbers of a likelihood of “trustworthiness” of data or meta‐data, even if the numbers are created by prestigious investigators or by a consensus voting in an organization or by an editorial team.

Admittedly, it seems very logical that by conducting a meta‐analysis on a set of RCTs judged to be “true”, one may derive an estimate of effectiveness we believe is “trustworthy”, given that a choice of potential biases has been identified and considered. Unfortunately, this idea is not so simple from a statistical perspective for at least two reasons.

The first reason is that effect estimations in most meta‐analyses leave out the element of random errors between the studies, e.g., as a reflection of small sample size and methodological heterogeneity combined with multiple testing. One may approximate that seemingly conclusive meta‐analyses become inconclusive (Brok, Thorlund, Wetterslev, & Gluud, 2009) after applying statistical methods termed recursive cumulative meta‐analysis (Ioannidis, Contopoulos‐Ioannidis, & Lau, 1999) or trial sequential analyses (TSA) (Wetterslev, Thorlund, Brok, & Gluud, 2008). Only a distinct minority of Cochrane SRs include TSA‐analyses. Hence, “trusted evidence” may not be so trustworthy after all, even if it comes from the Cochrane Collaboration, which emphasizes once again that in science, one may never prove anything, but rather one can disprove theories with a precise (low) level of probability.

Secondly, the practice of appraising only “quality‐trials” and stratifying meta‐analyses according to perceived bias has been criticized for at least two decades. Already in 1999, a group of authoritative epidemiologists used regression models to examine whether the type of quality assessment scale being used affected the conclusions of meta‐analytic studies. Their advice from their findings was “..that the use of summary scores to identify trials of high quality is problematic” (Jüni, Witschi, Bloch, & Egger, 1999). Twenty years later, another group of authoritative epidemiologists question why this practice is still maintained in Cochrane SRs since “stratification by quality leads to a form of selection bias, i.e., collider‐stratification bias, and should be avoided” in favor of other approaches (Stone et al., 2019).

A critical take‐home message is that some SRs present only amalgamated facts, while others also give their interpretations of the facts. These interpretations are invariably primed by their authors' theories, values, and ideologies (Wieringa, Engebretsen, Heggen, & Greenhalgh, 2018). It takes a trained mindset to perceive the almost imperceptible border between these two types of SRs. Proponents of compiling SRs that fit the first category can argue that non‐content experts can write adequate SRs and that even in some circumstances, avoiding content experts as co‐authors can be an advantage (Gøtzsche & Ioannidis, 2012). Proponents of complementing effectiveness with particular dimensions such as harm, or equity, or health economy elements will tend to both present and interpret the facts relative to these elements, which introduces a risk of introducing authors' biases. Notoriously controversial, are claims of the underreporting of side effects and risks of harm associated with interventions.

SRs that follow the existing minimum requirements for publishing results befitting the study design (https://www.equator‐network.org/) has helped tremendously for more efficient reporting and reading. I surmise that an SR reported according to the PRISMA format (Moher, Liberati, Tetzlaff, Altman, & The PRISMA Group, 2009) should be comparable with any Cochrane SR upon evaluation using AMSTAR (A MeaSurement Tool to Assess systematic Reviews) (Shea et al., 2007). Unfortunately, all SRs have shortcomings by default, including both PRISMA‐format and Cochrane SRs. The reason is that they are based only on what has been synthesized and published, and not on all data that have been recorded in a clinical study. I.e., there are always risks of potential outcome reporting biases in primary studies. Multiple articles identify incongruences between study intentions described in pretrial repositories and outcomes presented in final publications. Hence, there is a growing recognition that the current lack of open access to clinical study reports, and especially those involving effects of drug interventions, remain a barrier to provide unbiased evaluations. (Hodkinson et al., 2018). Perhaps the Cochrane Collaboration may re‐establish a reputation of impartiality and fairness by championing open access to clinical study reports as a basis for Cochrane SRs.

Regardless, a strategy that gives an impression of the charity becoming some sort of a moneymaking enterprise that rival “competitors” should be abandoned. It is astonishing that a statement on www: “Anyone who produces, or who finds a way to make systematic reviews more digestible and more relevant to the audience, is in competition with Cochrane” is attributed to the CEO of the charity. In contrast, my persuasion is that most health care providers and patients would like the charity to work together with and not compete with “anyone” for the betterment of health care.
